# The effect of experimentally induced sedentariness on mood and psychobiological responses to mental stress

**DOI:** 10.1192/bjp.bp.114.150755

**Published:** 2016-03

**Authors:** Romano Endrighi, Andrew Steptoe, Mark Hamer

**Affiliations:** **Romano Endrighi**, PhD, Department of Epidemiology & Public Health, University College London, UK and Department of Medical & Clinical Psychology, Uniformed Services University of the Health Sciences, Bethesda, Maryland, USA; **Andrew Steptoe**, DPhil, Department of Epidemiology & Public Health, University College London, UK; **Mark Hamer**, PhD, National Centre for Sport and Exercise Medicine, Loughborough University, UK

## Abstract

**Background**

Evidence suggests a link between sedentary behaviours and depressive symptoms. Mechanisms underlying this relationship are not understood, but inflammatory processes may be involved. Autonomic and inflammatory responses to stress may be heightened in sedentary individuals contributing to risk, but no study has experimentally investigated this.

**Aims**

To examine the effect of sedentary time on mood and stress responses using an experimental design.

**Method**

Forty-three individuals were assigned to a free-living sedentary condition and to a control condition (usual activity) in a cross-over, randomised fashion and were tested in a psychophysiology laboratory after spending 2 weeks in each condition. Participants completed mood questionnaires (General Health Questionnaire and Profile of Mood States) and wore a motion sensor for 4 weeks.

**Results**

Sedentary time increased by an average of 32 min/day (*P* = 0.01) during the experimental condition compared with control. Being sedentary resulted in increases in negative mood independent of changes in moderate to vigorous physical activity (ΔGHQ = 6.23, ΔPOMS = 2.80). Mood disturbances were associated with greater stress-induced inflammatory interleukin-6 (IL-6) responses (β = 0.37).

**Conclusions**

Two weeks of exposure to greater free-living sedentary time resulted in mood disturbances independent of reduction in physical activity. Stress-induced IL-6 responses were associated with changes in mood.

Extensive research has shown beneficial effects of physical activity on mental health^1,2^ although little is known about the role of sedentary behaviour. Sedentary behaviour does not simply reflect the bottom end of the physical activity continuum, but is now considered as an independent domain. Sedentary behaviour may be defined as any activity characterised by a low energy expenditure (⩽1.5 metabolic equivalents) in a sitting or reclining posture. It includes behaviours such as sitting and watching television or other screen-based activity including using computers for professional or entertainment purposes.^3,4^

Sedentary behaviour is increasingly recognised as a risk factor for cardiovascular diseases and metabolic disorders independent of physical activity.^5–7^ Sedentary behaviour such as television or screen-based entertainment time has also been prospectively associated with depressive symptoms.^8–10^ For example, in a large, 10-year follow-up study, time spent watching television was associated with an higher risk of depression independent of physical activity.^10^ In another prospective study, sitting for more than 42 h per week was associated with a 31% (95% CI 1.01–1.68) increased risk of developing a mental disorder compared with sitting for less than 10.5 h per week.^11^

To date, all of the evidence has been generated from epidemiological studies which cannot establish causality and leaves open the possibility that unmeasured variables may confound the findings. Additionally, studies have relied on self-report measures of sedentary time. Self-report suffers from recall bias and social desirability that likely result in underreporting of sedentary time.^12^

The mechanisms underlying the effect of sedentary time on mental health are not completely understood. Since inflammatory markers including interleukin-6 (IL-6), C-reactive protein (CRP) and tumor necrosis factor alpha (TNF-α) are implicated in depression and mood disorders,^13,14^ and inflammatory stimuli can cause transient mood disturbances in healthy individuals,^15^ a systemic inflammatory process may underlie the association of sedentary time with mood. Indeed, some studies have reported cross-sectional associations between inflammatory markers and sedentary time.^9,16–18^

Sedentary time may also contribute to a heightened risk of cardiovascular disease through an exaggerated or sustained cardiovascular and inflammatory response to stress. Heightened autonomic responses to mental stress are cross-sectionally and prospectively associated with biological risk factors including hypertension, coronary atherosclerosis and intima media thickness.^19–22^ Psychobiological responses to acute stress may be clinically relevant as they index an individual's typical response to daily stressors. Several mechanisms including central nervous system activation, immune cells redistribution and non-immune cell pathways are thought to mediate the effect of psychosocial stress on concentrations of inflammatory markers in the blood. One relevant mechanism is activation of transcription factors, since nuclear factor κB (NF-κB) DNA-binding activity in peripheral blood mononuclear cells is increased in response to acute stress.^23^ The activation of this transcription factor is thought to be responsible for triggering inflammatory gene expression^24^ and the release of inflammatory markers into the circulation. Chronic or sustained activation of this pathway by psychosocial stress over sustained time periods may result in dysregulation, which in turn may lead to a systemic inflammatory activity^14^ with adverse health consequences.

It is therefore plausible to hypothesise that sedentary individuals may be hyper-responsive to stress. This could be an important mechanism through which sedentary behaviour influences cardiovascular risk factors. In addition, pro-inflammatory responses to acute mental stress are known to be exaggerated in individuals with depressive symptoms possibly contributing to enhancing systemic inflammation and therefore mood disturbance. However, we are not aware of any study that has experimentally examined the effect of sedentary time on psychophysiological responses. Likewise, we are not aware of any study that has experimentally investigated the effect of sedentary time on mood. Such a study may offer strong support to the observational evidence linking sedentary time with depression and risk factors.

The aim of this study was to experimentally manipulate sedentary time under free-living conditions in a cross-over randomised fashion and examine the effect of this manipulation on mood symptoms and psychobiological responses to acute mental stress. It was hypothesised that: (a) in healthy participants, mood disturbance and psychological distress will develop after 2 weeks of a free-living sedentary intervention compared with 2 weeks of regular activity (control condition) independent of changes in measured physical activity; (b) psychobiological responses to acute mental stress will be greater following the sedentary condition compared with the control condition independent of changes in measured physical activity; and (c) changes in negative mood or distress post-intervention will be associated with greater pro-inflammatory responses to acute mental stress.

## Method

### Participants and study design

**Fig. 1:**
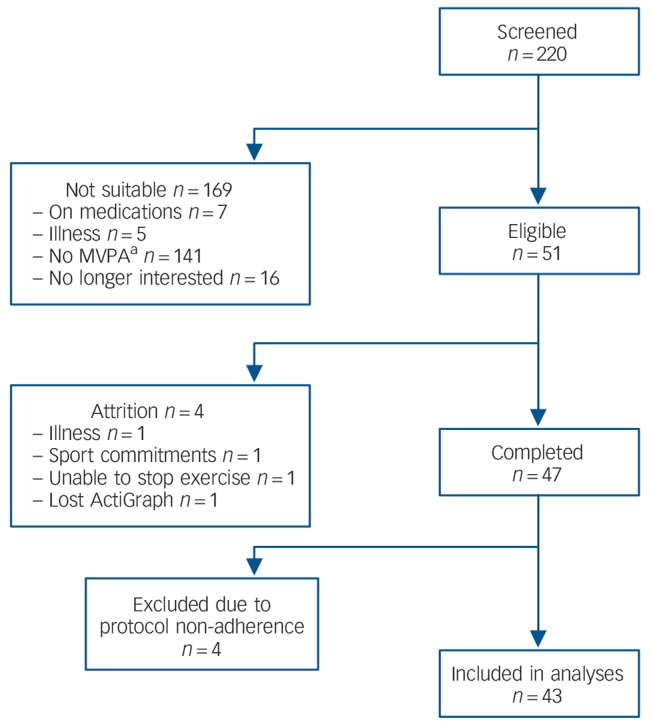
Diagram of participants' recruitment process. a. Not meeting study criteria for moderate and vigorous physical activity (MVPA) levels.

Based on prior evidence from our group,^25^ we anticipated small to medium intervention effects; thus, for a within-participant design (α = 0.05 and [1–β] = 0.80) a sample size of *n* = 46 was calculated. Fifty-one participants were recruited from the University of London student and staff population between February 2008 and December 2009 for a study examining physical activity and health. Participants were tested in the psychophysiology laboratory after 2 weeks of free-living sedentary behaviour (sedentary condition) and 2 weeks of normal activity (control condition) in a randomised, cross-over manner. Inclusion criteria were: being regularly active (moderate and vigorous physical activity (MVPA) at least three times a week for 1 h each session), aged between 18 and 35 years, not on any regular medication, non-smokers and a body mass index (BMI) of ⩾19 and ⩽25 kg/m^2^. Eligibility criteria were ascertained by telephone screening interviews prior to study enrollment (Fig. 1).

Ethical approval was granted by the Joint University College London/University College London Hospital Research Ethics Committees on the Ethics of Human Research and all participants signed an informed consent form prior to study enrollment.

### Procedure

At time 0, participants were equipped with an accelerometer (ActiGraph) and randomised to a control condition or a sedentary condition. Following completion of the first condition (+2 weeks), participants reported back to the laboratory to complete mood assessments and for a psychophysiological testing session. Participants were required to switch condition and return to the laboratory (+4 weeks) for repeated mood assessments and psychophysiological testing. All participants were instructed to refrain from vigorous physical activity and from drinking alcoholic beverages the night before scheduled testing appointment, and to have a light, low-fat meal no later than 3 h before the appointment.

A research nurse masked to the study's hypotheses provided participants with a letter of condition allocation. The letter also contained instructions and requirements for each condition. In the sedentary condition, participants were instructed to replace any daily structured or unstructured form of physical activity by being sedentary, and were encouraged to be sedentary as much as possible. In the control condition, participants were instructed to maintain their habitual levels of daily activity. Participants were required to wear the ActiGraph around their waist as instructed every day after waking and until bedtime, and only remove it briefly when showering or swimming.

### Measures

Anthropometric measures were obtained by a research nurse using standard protocol.

#### Mood outcomes

Psychological distress was assessed using the General Health Questionnaire (GHQ-28).^26^ It has four subscales (somatic symptoms, anxiety/insomnia, social dysfunction and depression) that measure the participant's current state of emotional distress as compared with the usual state. According to established norms,^27^ each scale has a possible score range of 0–21, and a total score (range 0–84) may be computed by adding up the subscales. Higher values indicate greater distress. Cronbach's α was 0.85 (control) and 0.90 (sedentary). Mood was assessed with the short version of the Profile of Mood States (POMS-SF).^28^ This question-naire has been used extensively in a variety of settings with healthy and clinical populations, and has good psychometric properties^29^ comparable to the original, longer version. The POMS-SF measures the following six dimensions of mood states (score ranges): tension-anxiety (0–24), vigor-activity (0–24), depression-dejection (0–32), fatigue-inertia (0–20), confusion-bewilderment (0–20) and anger-hostility (0–28). A negative mood mean score was also computed by adding the five negative mood subscales and subtracting vigor/activity (range 0–100), with higher scores reflective of greater negative affective states. Cronbach's α was 0.81 (sedentary) and 0.84 (control).

#### Adherence

ActiGraph GT1M (accelerometer, LLC, Pensacola, Florida, USA) was used as a manipulation check to assess adherence to study conditions and to compute the outcome variables sedentary time and MVPA. Raw data were computed into average counts per minute per day (CPM/d) of activity using the MAH/UFFE Analyzer version 1.9.0.3 (Cambridge, UK). A wear time of 10 h per day was considered valid. Any continuous 60-minute period of zero counts was considered as non-wear time. Cut-offs used to categorise intensity domains were based on Matthew^30^ and are as follow: sedentary <190 count.min^−1^, light activity 190–573 count.min^−1^, moderate activity 574–2099 count.min^−1^, vigorous and very vigorous activity 2100 count.min^−1^ and above. Sedentary time was computed as daily total wear time minus total daily active time,^12^ MVPA was computed by adding the moderate to very vigorous intensity activity categories.^25^

#### Acute stress

Mental stress was elicited with two 5-minute, standardised stress tasks administered under time pressure which have been previously described.^31^ In the mirror tracing task, participants were instructed to trace around a marked contour of a star with an electronic pen while looking at the star's own reflection in a mirror. In the public speaking task, participants were presented with two scenarios in which they were required to defend themselves to avoid redundancy or being wrongly accused of theft. Participants were told that the performance would be video recorded and rated by experts. Standard written instructions were provided prior to each task. Mental stress testing was carried out either in the morning (starting 10.00 h) or in the afternoon (starting 12.00 h), but each participant was tested at the same time in the control and sedentary conditions.

#### Inflammatory markers

To measure inflammatory responses to mental stress blood was sampled at the end of the baseline period (rest) and 45 min post stressors. Peripheral blood was drawn from the antecubital vein of the forearm into EDTA-coated vacutainers for plasma and serum separator tubes for serum. EDTA samples were immediately centrifuged, whereas serum samples were left to clot at room temperature for 30 min before centrifugation. All samples were centrifuged at 1246 × *g* for 10 min at room temperature. The resulting supernatant was removed and immediately frozen in a −80°C professional freezer in 0.50 ml aliquots before biochemical assay. Samples were stored for a maximum of 2.5 years before assay.

Plasma IL-6 was analysed in duplicate using a high sensitivity ELISA (limit of detection of 0.016 pg/ml; intra- and inter-assay coefficients of variation of 7 and 7.20% respectively) supplied by R&D (R&D System, Oxford, UK). To measure low-grade systemic inflammation, CRP was assessed at baseline only using an ELISA kit supplied by R&D with a limit of detection of 0.005 ng/ml and with intra- and inter-assay coefficients of variation of 5.50 and 6.50% respectively. Concentrations of IL-6 and CRP were determined with a microplate reader (Molecular Devices, UK) using the SoftMax Pro 5 software. The software generates standard curves by reducing raw absorbance data to a linear log/log parameter (IL-6) and four parameter logistic curves (CRP) according to respective protocols (R&D System, Oxford, UK).

#### Cortisol

Saliva samples were obtained at the end of the baseline period, immediately after stress, 20 and 45 minutes post-stress. Samples were assayed in duplicate at the Kurume University in Japan using a cortisol enzyme immunoassay kit by Salimetric. The correlation of serum cortisol with salivary cortisol is *r* = 0.91 as reported by the manufacturer.

#### Cardiovascular

Beat by beat blood pressure was monitored continuously during the mental stress sessions with a Finometer Pro (Finapres Medical System, The Netherlands) attached to the middle finger of the non-dominant arm via a small cuff. Heart rate was measured continuously using an ActiHeart (Cambridge, Neurotechnology) device attached to the participant's chest with electrocardiogram electrodes.

### Analytic strategy

Adherence to the sedentary intervention protocol was established by comparing objectively derived sedentary time data in the control and sedentary conditions using related *t*-test. Four participants did not provide valid ActiGraph data either because of failure to wear the device or suboptimal wear time and were thus removed from analyses. Mixed analysis of variance (ANOVA) models with condition (sedentary and control) as within-participant factor, and order of study condition allocation as fixed factor tested the effect of sedentary behaviour on the GHQ and POMS scores. Change scores (mean difference from control to sedentary) were computed for the GHQ, POMS, sedentary time and MVPA, and were used in regression models to test whether changes in sedentary time were associated with changes in mood, and if this association was independent of changes in objective MVPA. Changes in basal level of inflammatory markers were tested using related *t*-tests.

Cardiovascular stress responses were averaged over 5-minute trials to yield a baseline value (mean of last 5 min of the rest period), a stress value (mean of speech and mirror task) and a recovery value (mean of the last 5 min of the recovery period). Main effects and interaction effects were determined using mixed ANOVA models with condition (sedentary and control) and trial (baseline, stress and recovery) as within-participants and order of condition allocation as a covariate (to control for a possible order effect). Significant interaction effects were examined by comparing stress reactivity (stress–baseline values) and recovery scores (recovery values–baseline) using paired *t*-test. We also computed an area under the curve (AUC) for cortisol^32^ to index total hormonal output during mental stress and compared it across experimental conditions. Greenhouse–Geisser correction for degrees of freedom is presented where appropriate. Cortisol and IL-6 data were logarithmically transformed to normalise the distribution but natural values are presented to aid interpretation.

To examine associations between changes in mood and psychological distress after the intervention and IL-6 acute stress responses, hierarchical linear regression was used with GHQ or POMS change scores as predictors and the IL-6 stress response in the sedentary condition as outcome. Models were adjusted for the IL-6 stress response during control condition testing. Results are presented as β-coefficients and change in *R*^2^ (Δ*R*^2^), and *P* values. Associations were illustrated by displaying the mean IL-6 stress response of people in the lower, middle and higher tertiles of mood change.

## Results

### Sample characteristics

Table 1 shows the characteristics of the sample at study entry. Participants were normotensive with BMI values in the normal range. As expected, males had higher resting systolic blood pressure than females. Participants excluded from the analysis (*n* = 4) did not differ from the main sample in age, BMI, resting blood pressure and resting inflammatory markers (all *P*>0.10).

**Table 1:** Descriptive characteristics of the sample at study entry (*n* = 43)

Variable	Male (*n* = 24)	Female (*n* = 19)	*P*
Age, years	23.86 (4.71)	25.73 (4.24)	0.18

BMI, kg/m^2^	23.01 (2.34)	23.30 (2.47)	0.70

SBP, mmHg	115.29 (10.98)	108.26 (9.26)	0.03

DBP, mmHg	63.41 (7.38)	67.84 (8.63)	0.07

HR, bpm	66.61 (10.68)	67.75 (12.82)	0.75

BMI, body mass index; SBP, systolic blood pressure; DBP, diastolic blood pressure; HR, heart rate.

Values are means (s.d.).

### Sedentary time

#### Intervention effect

Analysis revealed a significant main effect of time for sedentary time (*F*(1, 41) = 4.20, *P* = 0.04). Pairwise comparison revealed that sitting time increased by an average of 31.49 m/d (s.e. = 12.13, *P* = 0.01) during the sedentary condition. This effect was independent of a possible condition allocation order effect (*F*(1,41) = 0.02, *P* = 0.88). There was also a main effect of time for MVPA (*F*(1,41) = 33.69, *P*⩽0.0001). Adjusted comparisons indicated that MVPA level decreased by 36.86 m/d (s.e. = 4.73, *P*⩽0.0001) from control to the sedentary condition (Table 2).

**Table 2:** Summary of changes in ActiGraph measured daily activity (*n* = 43)

ActiGraph variable	Control	Sedentary	*P*
Sedentary time, min/day	575.47 (7.21)	606.95 (85.95)	0.01

MVPA, min/day	139.65 (39.26)	102.79 (34.73)	<0.001

Light activity, min/day	82.49 (21.34)	71.11 (24.17)	<0.001

MVPA, moderate and vigorous physical activity.

Values are means (s.d.).

### Mood outcome effects

There was a main effect of time for GHQ score (*F*(1,41) = 10.23, *P* = 0.003). Condition allocation order (*F*(1,41) = 0.002, *P* = 0.96) did not moderate this effect. Adjusted comparisons showed a significant mean increase of 6.23 (s.e. = 1.59, *P*⩽0.0001) GHQ points in the sedentary condition. There was also a main effect of time for the POMS score (*F*(1,41) = 16.51, *P*⩽0.0001). Negative mood increased by 2.80 (s.e. = 0.55, *P*⩽0.0001) points. The order to which participants were assigned to conditions was not related to the POMS (*F*(1,41) = 0.54, *P* = 0.46). Table 3 summarises the results for the subscale scores of the GHQ and the POMS across conditions. The sedentary intervention resulted in increases in distress and negative mood across all subscales except the depression dimension of the GHQ.

**Table 3:** Summary of the effect of the sedentary intervention on mood symptoms (POMS) and psychological distress (GHQ) (*n* = 43)

Outcome	Sedentary	Control	t _(df)_	*P*
GHQ anxiety/insomnia	5.69 (4.07)	3.65 (2.86)	3.07 _(42)_	0.004

GHQ depression	0.97 (2.47)	0.90 (2.34)	0.14 _(42)_	0.88

GHQ somatic symptoms	5.88 (3.92)	3.25 (2.20)	4.10 _(42)_	<0.001

GHQ social dysfunction	7.86 (2.17)	6.37 (1.75)	4.62 _(42)_	<0.001

POMS tension/anxiety	7.58 (4.34)	5.44 (3.52)	3.10 _(42)_	0.003

POMS vigor/activity	8.07 (3.90)	14.11 (4.12)	−7.07 _(42)_	<0.001

POMS depression/dejection	3.65 (3.64)	2.53 (3.19)	1.96 _(42)_	0.05

POMS fatigue/inertia	8.30 (5.19)	4.95 (4.38)	3.78 _(42)_	<0.001

POMS confusion/bewilderment	7.41 (4.22)	4.93 (3.29)	3.89 _(42)_	<0.001

POMS anger/hostility	4.79 (4.29)	3.14 (3.75)	3.06 _(42)_	0.004

GHQ, General Health Questionnaire; POMS, Profile of Mood Scale.

Values are means (s.d.).

### Associations between sedentariness and mood outcomes

The increase in sedentary time was significantly associated with the POMS negative mood score (β = 0.32, *R*^2^ = 0.10, *P* = 0.03), and this association persisted after controlling for changes in MVPA (β = 0.32, *P* = 0.05). MVPA was not associated with the POMS (β = −0.003, *P* = 0.98). No significant associations emerged between GHQ scores and changes in sedentary time (β = 0.25, *R*^2^ = 0.06, *P* = 0.1) or in MVPA (β = 0.08, *P* = 0.62).

### Mental stress responses

#### Cardiovascular

Participants showed comparable levels of subjective involvement in carrying out the stress tasks across the testing sessions (control: *M* = 5.21, s.d. = 1.12; sedentary: *M* = 5.26, s.d. = 1.07, *t*(42) = 0.38, *P* = 0.70). There was a main effect of time for systolic blood pressure (SBP) (*F*(2,82) = 64.04, *P*⩽0.0001) and diastolic blood pressure (DBP) (*F*(2,82) = 77.96, *P*⩽0.0001) indicating that blood pressure increased significantly in response to stress returning towards baseline levels by the end of the stress protocol (quadratic time effect: SBP = *F*(1,41) = 87.29, *P*⩽0.0001; DBP = *F*(1,41) = 105.16, *P*⩽0.0001). The condition × time interaction was not significant for SBP (*F*(2,82) = 2.45, *P* = 0.09) or for DBP (*F*(2,82) = 1.53, *P* = 0.22), suggesting no differences in stress reactivity or recovery across conditions.

**Fig. 2:**
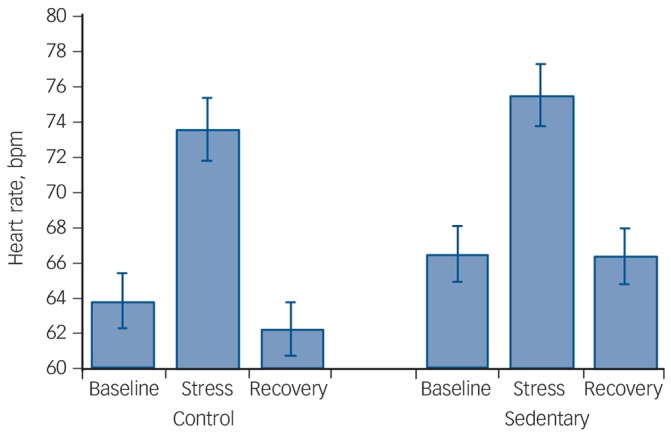
Acute stress-induced heart rate response (*n* = 43). The heart rate response to mental stress after 2 weeks of usual activity (control condition) and after 2 weeks of sedentary activity (sedentary condition) in a randomised, cross-over design. Stress reactivity (*P* = 0.49) or stress recovery (*P* = 0.12) was not significantly different across experimental conditions.

Analyses of heart rate responses revealed a main effect of time (*F*(1.58, 64.80) = 48.72, *P*⩽0.0001), suggesting an increase in heart rate in response to stress with values returning to below baseline levels by the end of the protocol (quadratic time effect *F*(1,41) = 66.27, *P*⩽0.0001). There was also a significant condition × time interaction (*F*(2,82) = 4.53, *P* = 0.01) indicative of a different pattern of responses (Fig. 2). Further examining this interaction by comparing stress reactivity and stress recovery values revealed no significant differences in either reactivity (*P* = 0.49) or recovery (0.12), suggesting that the interaction may be accounted for by higher baseline values in the sedentary condition (mean 66.65 (s.d. = 10.17) bpm *v.* mean 63.98 (s.d. = 11.62) bpm) rather than higher stress reactivity.

#### Neuroendocrine

Salivary cortisol concentration during control condition averaged 2.89 (s.d. = 1.80) nmol/l at baseline, 3.19 (1.77) nmol/l immediately after stress, 2.42 (1.49) nmol/l 20 min post-stress and 2.12 (1.37) nmol/l 45 min post-stress. During the sedentary condition, cortisol concentration averaged 3.37 (s.d. = 2.72) nmol/l at baseline, 3.66 (2.78) nmol/l immediately after stress, 3.21 (2.25) nmol/l 20 min post-stress and 2.49 (1.52) 45 min post-stress. There was a significant main effect of time (*F*(3,123) = 8.80, *P*⩽0.0001) but there was no significant condition × time interaction (*F*(3,123) = 1.60, *P*= 0.19), indicating that the stress protocol induced a significant response but with no significant differences in this response across conditions. Total cortisol output (computed as AUC) was not significantly different across sedentary and control conditions respectively (*M* = 358.60, s.d. = 207.59 nmol/l/m *v. M* = 311.92, s.d. = 150.90 nmol/l/m; *t*(42) = 1.50, *P* = 0.14).

#### Inflammatory

There was wide variation in the IL-6 reactivity to acute mental stress. On average, IL-6 increased by 0.032 pg/ml (range −1.07 to 1.13 pg/ml) from baseline to 45 min post-stress at control testing, and by 0.042 pg/ml (range −0.28 to 0.76 pg/ml) at sedentary testing. There was no main effect of time (*F*(1,41) = 0.29, *P* = 0.59) and no condition × time interaction (*F*(1,41) = 1.44, *P* = 0.23), indicating no differences in reactivity across the study conditions.

### Inflammation and mood outcomes

#### Basal inflammatory activity

Increased sedentary time did not influence resting levels of CRP (0.95 (s.d. = 1.65) mg/L control condition *v.* 1.10 (s.d. = 1.71) mg/L sedentary condition, *P* = 0.38) or IL-6 level (1.01 (s.d. = 1.34) pg/ml control condition *v.* 1.00 (s.d. = 1.74) pg/ml sedentary condition, *P* = 0.60).

#### Associations between mood and inflammatory stress reactivity

**Fig. 3:**
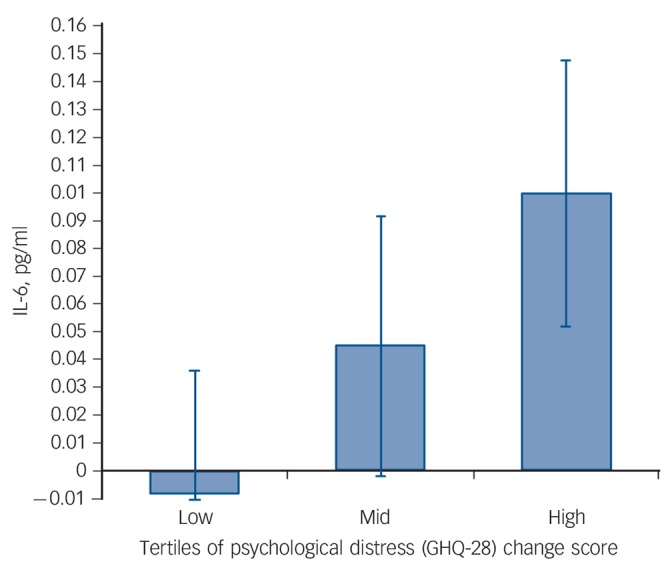
Association of interleukin-6 (IL-6) stress response and sedentary-induced changes in General Health Questionnaire (GHQ) score. The IL-6 acute stress response at sedentary testing in individuals with low, mid and high change in the GHQ score following the sedentary intervention. Values are adjusted for the IL-6 stress response during control testing (*n* = 43).

The pro-inflammatory IL-6 response to acute stress at the sedentary condition was positively associated with the GHQ change score (β = 0.37, *P* = 0.01, Δ*R*^2^ = 0.14) after adjusting for the IL-6 response at control testing. There was also a borderline significant association of the IL-6 response with the POMS change score (β = 0.28, *P* = 0.06, Δ*R*^2^ = 0.08) after adjustment for the IL-6 response at control. Neither the GHQ (β = 0.05, *P* = 0.75) nor the POMS (β = 0.02, *P* = 0.90) was associated with the IL-6 stress response in the control condition. These data indicate that greater mood disturbance following 2 weeks of increased free-living sedentary time was associated with an heightened IL-6 release in response to acute stress independent of the IL-6 response at control stress testing (Fig. 3).

## Discussion

This is the first experimental study to test the effect of manipulating free-living sedentary time on mood outcomes and psychobiological responses to mental stress. Furthermore, this study examined whether changes in mood post-intervention were associated with pro-inflammatory responses to mental stress independent of response at control testing. We induced approximately 7.5 h of additional free-living sedentary time during the experiment as confirmed by objective measurement using an accelerometer device. Our prediction that sedentary time would result in negative mood symptoms was supported. Increased sedentary time was associated with negative mood which was independent of changes in objectively measured MVPA. Therefore, using an experimental design we demonstrated a link between sedentary time and the development of negative mood and psychological distress, which largely supports the findings from observational studies.

We measured a wide range of cardiovascular (blood pressure and heart rate), neuroendocrine (cortisol) and inflammatory (IL-6) markers in response to acute mental stress, but contrary to our hypothesis there was limited evidence that sedentary behaviour resulted in heightened psychophysiological responses. There was no difference in reactivity to or recovery from acute stress in the sedentary condition compared with the control condition in the measure assessed. Although we found a condition × time interaction for heart rate suggesting greater responses in the sedentary condition (Fig. 2), a closer examination of the interaction indicated that the effect may be accounted for by higher baseline heart rate values in the sedentary condition stress testing rather than greater reactivity.

One reason that could explain the null effects is that the duration of the intervention and the magnitude in changes to sedentary behaviour might not have been sufficient to induce such marked stress-induced physiological changes in this healthy sample. Sedentary time may lead to vascular and metabolic adaptations including changes in insulin sensitivity and accumulation of visceral adipose tissue^33^ that may precede changes in stress reactivity or stress recovery. These changes may emerge in response to chronic exposure to a sedentary lifestyle.

Basal levels of the inflammatory markers CRP and IL-6 did not change following 2 weeks of increased sedentary time compared with control. This is not consistent with previous epidemiological work that showed an association between basal circulating CRP and television viewing (as an index of sedentary time).^9^ Other recent work has shown favourable effects of acutely breaking up prolonged sitting on the expression of various genes linked to inflammatory responses.^34^

Our hypothesis that mood disturbances or psychological distress following sedentary intervention would be associated with pro-inflammatory responses was supported. We showed that negative mood was associated with the IL-6 pro-inflammatory stress response independent of the IL-6 response during control. Individuals with greater mood disturbances measured with the POMS following sedentary time displayed an heightened IL-6 response. There was also a near-significant association of psychological distress measured with the GHQ and pro-inflammatory IL-6 responses. This suggests that the transient mood disturbances induced by being sedentary may interact with acute stress to enhance pro-inflammatory reactivity. This finding can be interpreted in relation to the literature on depressive symptoms and acute inflammatory stress reactivity. It has been shown that depressed mood at the subthreshold level measured continuously^35^ or as a binary variable^36,37^ was associated to greater pro-inflammatory IL-6 responses to mental stress independent of BMI and resting levels. Sedentary behaviour may therefore contribute to negative mood by enhancing inflammatory responses to stress leading to an upregulation of the inflammatory signalling pathway and enhanced vulnerability to mood disturbances. However, at present this hypothesis can only be speculative since to address this question large experimental studies with adequate follow-up periods would be required. Other mechanisms may also be involved. For example, displacement of physical activity with passive sedentary activities such as television viewing might encourage social isolation, known to be linked with depression.^38^

### Strengths and limitations

This study has some limitations. Our sample included habitually active and healthy individuals and the findings may not generalise to the general population. In particular, caution should be employed in generalising the findings of this study to individuals with clinical depression or clinical levels of psychological distress or indeed to individuals with chronic illnesses for whom being sedentary may be a consequence rather than a cause of depression. Blood samples were obtained at baseline and 45 min post-stress only but evidence suggest that IL-6 may continue to increase up to 90 min post-stressor.^39^ Had we employed a longer post-stress recovery period and further sampling we might have uncovered effects that were not apparent using the current protocol. Although IL-6 seems to be reliably activated by mental stress and to play an important role in mood disorders,^14^ other cytokines including TNF-α, IL-1ra and IL-1β appear to be relevant in the association of sedentary behaviour and mood but were not assessed in this study. The method used to assess sedentary time in this study has some limitations. The ActiGraph quantifies time spent in different intensities of activity by summing time above and below specified count thresholds which have to be chosen according to the population of interest.^40^ ActiGraph wear time in this study was about 12 h/day thus we cannot account for non-wear periods or potential daytime naps. Nevertheless, wear time did not differ between experimental conditions. Contextual information on sedentary time was not collected, which may be important in relation to mental health,^41^ nor was any familial history of risk factors such as coronary heart disease or depressive disorders which may potentially affect stress reactivity.^42^ Strengths of the study include the use of a wide range of stress-induced measures and the cross-over study design that is less prone to inter-individual confounding. In addition, a notable strength of the study is the objective assessment of sedentary time and activity using an accelerometer rather than relying on self-report as is the case in the majority of studies of sedentariness and mental health.

### Implications

The findings of this study have some important implications. Sedentary time does not simply reflect the lower scale of the physical activity continuum but represent a risk factor for mental health in its own right. Therefore, efforts should also be devoted to modifying sedentariness as well as promoting physical activity especially in populations more at risk of mental health problems.

In summary, we have shown for the first time that experimentally manipulating free-living sedentary time resulted in robust increases in psychological distress and mood disturbance, and these changes were associated with greater inflammatory responses to acute stress. The link between sedentary behaviour and mood may therefore partly be driven by repeated or sustained inflammatory responses to daily stressors, but further research is required to tease apart whether mood disturbances drive inflammatory responses or vice versa.
